# Authentication of Geographical Origin in Hainan Partridge Tea (*Mallotus obongifolius*) by Stable Isotope and Targeted Metabolomics Combined with Chemometrics

**DOI:** 10.3390/foods10092130

**Published:** 2021-09-09

**Authors:** Jiashun Fu, Hai-Dong Yu, Long Wu, Chenghui Zhang, Yong-Huan Yun, Weimin Zhang

**Affiliations:** 1 School of Food Science and Engineering, Hainan University, Haikou 570228, China; fujiashun1998@163.com (J.F.); haidongyu@yeah.net (H.-D.Y.); longquan.good@163.com (L.W.); zchlm@163.com (C.Z.); zhwm1979@163.com (W.Z.); 2 Key Laboratory of Food Nutrition and Functional Food of Hainan Province, Haikou 570228, China

**Keywords:** stable isotope, polyphenols, chemometrics, metabolomics, geographical origin, tea

## Abstract

Partridge tea (*Mallotus oblongifolius* (Miq.) Müll.Arg.) is a local characteristic tea in Hainan, the southernmost province of China, and the quality of partridge tea may be affected by the producing areas. In this study, stable isotope and targeted metabolomics combined chemometrics were used as potential tools for analyzing and identifying partridge tea from different origins. Elemental analysis—stable isotope ratio mass spectrometer and liquid chromatography-tandem mass spectrometrywas used to analyze the characteristics of C/N/O/H stable isotopes and 54 chemical components, including polyphenols and alkaloids in partridge tea samples from four regions in Hainan (Wanning, Wenchang, Sanya and Baoting). The results showed that there were significant differences in the stable isotope ratios and polyphenol and alkaloid contents of partridge tea from different origins, and both could accurately classify partridge tea from different origins. The correct separation and clustering of the samples were observed by principal component analysis and the cross-validated Q^2^ values by orthogonal partial least squares discriminant analysis (OPLS-DA) were 0.949 (based on stable isotope) and 0.974 (based on polyphenol and alkaloid), respectively. Potential significance indicators for origin identification were screened out by OPLS-DA and random forest algorithm, including three stable isotopes (δ^13^C, δ D, and δ^18^O) and four polyphenols (luteolin, protocatechuic acid, astragalin, and naringenin). This study can provide a preliminary guide for the origin identification of Hainan partridge tea.

## 1. Introduction

Tea is one of the most popular non-alcoholic beverages consumed by more than two-thirds of the world’s population because of its attractive aroma, refreshing mouth feel, and multiple health benefits [1]. Planting environments, geographical conditions, and processing methods are the important factors that directly affect the quality of tea [2,3]. The higher quality and better reputation of tea with a higher price is understandable. However, because the sensory qualities of the same kind of tea or different kinds of tea are similar, consumers alone cannot correctly judge the quality of tea. This has enabled some unscrupulous businesses to make shoddy or adulterated products in their productions for achieving the purpose of making huge profits. Partridge tea (*Mallotus oblongifolius* [Miq.] Müll.Arg.) is a kind of substitute tea beverage with strong local characteristics in Hainan Province (the southernmost province of China), which has important medicinal and economic value and has long been known as “glossy ganoderma” [4]. Partridge tea is distributed all over Hainan island, where Wanning and Wenchang are the most famous producers [5]. Therefore, merchants may mislabel their products and replace Wanning and Wenchang partridge tea from other regions so as to obtain higher benefits. Therefore, in order to protect consumers from fraud and maintain the reputation of the business, it is very important to develop a scientific and accurate method to analyze and verify the authenticity of partridge tea products.

Isotope characteristics are transferred from the growth environment, which produce different stable isotope characteristics in organisms, and can reflect the information of biological growth environment and its adaptability to environmental changes. They depend on variables such as distance from the ocean, climate, temperature, altitude, basic geology, and soil composition [6]. In recent years, stable isotope ratios have been increasingly used for geographical traceability and identification of tea, such as the identification of four kinds of Chongqing tuo tea [7] and the origin tracing of Guizhou green tea, Xihu Longjing, and green tea in various regions of China [2,8,9]. This technique is primarily based on the ratio of stable C/N/O/H isotopes relative to the geographic origin and plant metabolism. The values of δ^13^C are generally related to plant species due to the different carbon sequestration in different plants. However, geographical effects (different latitude, elevation, elevation, rainfall, water stress, and light) and management measures may also affect the efficiency of carbon dioxide sequestration, even within the same species [10]. The δ^15^N values reflect the nitrogen sources of plants; while they generally cannot directly reflect the geographical sources, different δ^15^N values can reflect the local soil properties and agricultural practices [11]. The δ D and δ^18^O values of plants are closely related to the local rainfall and geographical location, which can reflect the isotopic characteristics and climatic characteristics of the water consumed locally. Therefore, δ D and δ^18^O values are usually used as powerful indicators to distinguish the geographical origin [12,13].

If the geochemical or soil information between different regions is too similar, other parameters of the sample molecular composition level may also help to further distinguish geographical sources [14]. In particular, for tea, polyphenols and alkaloids are the most important bioactive components [15], which are closely related to various health functions, such as anti-cancer, cardiovascular protection, weight loss, anti-diabetes, and other benefits [16], and have always been the focus of tea research. In the chemical composition of tea, polyphenolic compounds account for the dominant part, accounting for approximately 25% to 35% of the dry weight [17]. The relative polyphenol and alkaloid components and contents of tea are closely related to the degree of fermentation, growth conditions, and geographical location [15,18], so we can identify the tea according to the differences among different teas. Related studies have also shown that polyphenols and alkaloids in tea has great potential in the identification and traceability. For example, Ning et al. successfully distinguished six kinds of tea from different origins (green tea, black tea, white tea, oolong tea, yellow tea, and black tea) by detecting caffeine, theanine, and six kinds of polyphenols in tea. The total identification rates of the training set and the prediction set were 94.13% and 92.31%, respectively [19]. Wang et al. used six kinds of tea polyphenols and an alkaloid to identify the geographical sources of oolong tea from three main producing areas in China (Fujian, Taiwan and Guangdong Province) [20]; Wu et al. identified four kinds of Chinese tea with different fermentation degrees (green tea, white tea, oolong tea, and black tea) by detecting tea polyphenols and alkaloids in fermented tea, and the correct rate of discrimination was as high as 97.8% [21]; Xin et al. used the differences of 75 chemical components (tea polyphenols, alkaloids, and theanine) in green tea to identify green tea from Yunnan and Hunan Provinces. The partial least squares model established by Xin et al. has a high accuracy rate of 98.61% [22]. Therefore, it was highly feasible to identify and trace the origin of tea based on the differences of its main chemical components.

At present, methods based on stable isotope ratio mass spectrometry (IRMS) and metabolomics have not been used to determine the correct geographic origin of partridge tea from different origins in Hainan. In this study, IRMS and liquid chromatography-tandem mass spectrometry (LC-MS/MS) were used to analyze the stable isotope ratios and the contents of polyphenols and alkaloids in partridge tea, and in combination with chemometrics, to analyze the differences and identify the partridge tea from different geographical origins. The isotope characteristics and major metabolite information of partridge tea from different origins were preliminarily explored, and the orthogonal partial least squares discriminant analysis (OPLS-DA) models were established for geographical origin prediction. The effective indicators for distinguishing partridge tea from different origins were screened out through OPLS-DA and random forest. This study provided a theoretical basis for the origin traceability of Hainan partridge tea and helped to promote the development of Hainan partridge tea.

## 2. Materials and Methods

### 2.1. Samples Preparation

**Figure 1:**
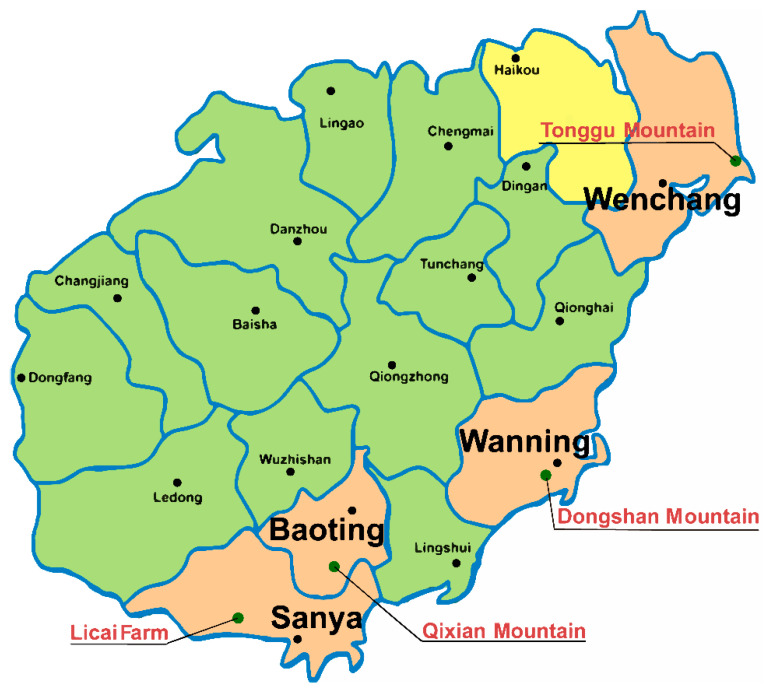
The specific producing areas of partridge tea samples in the southernmost province of China, Hainan, and the green dots in the figure are the sampling places.

The partridge tea samples used in the current study were collected from the major producing areas of partridge tea in Hainan Province (longitude 108°37′–111°03′, latitude 18°10′–20°10′). A total of 24 samples were separated into four groups, including Tonggu Mountain of Wenchang (WC, n = 6), Dongshan Mountain of Wanning (WN, n = 6), Qixian Mountain of Baoting (BT, n = 6), and Licai Farm of Sanya (SY, n = 6). All tea samples were randomly collected from tea gardens in the corresponding producing areas in May 2020, the collected samples were the fresh leaves of partridge tea tree, and the average age of the partridge tea trees was above 3 years. To ensure sample collection uniformity and repeatability, all tea leaves from each experimental group were obtained from five different regional parcels. The specific sampling locations are shown in Figure 1. After picking, the partridge tea was dried in the shade for 36 h, then crushed, sieved with 60 mesh sieves, packed in sealed bags, and dried in a dryer for later use. In addition, quality control (QC) samples were prepared by mixing all samples according to identical steps as the actual samples.

### 2.2. C/N/O/H Stable Isotope Amount Ratio Analysis

The C/N/O/H stable isotope ratio in partridge tea samples was determined by Vario Pyro Cube element analyzer (EA, ELEMENTAR, Langenselbold, Germany) combined with IRMS (Isoprime precision, ELEMENTAR, Langenselbold, Germany). The samples were dried at 70 °C for at least 24 h before the H/O measurement. Approximately 2.0 mg of partridge tea fine powder was weighed into a 6 × 4 mm tin box for δ^13^C and δ^15^N analysis, and approximately 1.2 mg of partridge tea fine powder was weighed into a 6 × 4 mm silver box for δ D and δ^18^O analysis. The environment was equilibrate to laboratory atmospheric conditions prior to δ D and δ^18^O analysis. The tin/silver box was folded and compressed out of air, then put into EA and connected with IRMS using an automatic sampler. Analytical conditions were as follows: high temperature furnace temperature of the EA was set at 1120 °C for δ^13^C analysis and 850 °C for δ^15^N analysis with a carrier gas flow of 230 mL/min; the pyrolysis furnace (for δ D and δ^18^O analyses) was set to 1450 °C with a carrier gas flow of 125 mL/min. The carrier gas was helium.

The final stable isotope ratio is calculated according to the following equation, which is represented by δ:

δ = [(R_sample_/R_standard_) − 1] (1)

where R_sample_ indicates the heavy/light isotope ratio (i.e., ^13^C/^12^C, ^15^N/^14^N, ^2^H/^1^H and ^18^O/^16^O) of the sample to be tested (partridge tea in this study), and R_standard_ expresses the heavy/light isotope ratio based on the national standard (Vienna Pee Dee Belimnite (VPDB) for carbon, atmospheric nitrogen (AIR) for nitrogen, and Vienna Standard Mean Ocean Water (VSMOW) for hydrogen and oxygen). The δ values are expressed in parts per thousand, in units of “per mil” (%).

Main materials for multi-point calibration of stable isotopes (EMA, Elemental Microanalysis, Okehampton, UK): B2155 (Protein, δ^13^C_VPDB_ = −26.98‰, δ^15^N_AIR_ = 5.94‰), B2174 (Urea, δ^13^C_VPDB_ = −36.59‰, δ^15^N_AIR_ = −2.20‰, B2203(EMA P1, δ^18^O_VSMOW_ = 20.99‰, δ^2^H_VSMOW_ = −25.30‰, B2205(EMA P2, δ^18^O_VSMOW_ = 26.88‰, δ^2^H_VSMOW_ = −87.80‰). Based on the instrument accuracy of stable isotope ratio measurement of reference substance, δ^13^C ≤ 0.13‰, δ^15^N value ≤ 0.12‰, δ D value ≤ 5.10‰, and δ^18^O value ≤ 1.88‰.

### 2.3. Polyphenols and Alkaloids Analysis

#### 2.3.1. Reagents and Instruments

Water was purified using an ultrapure water preparation system; HPLC grade acetonitrile and methanol were purchased from Fisher (Hampton, NH, USA); HPLC grade formic acid, acetic acid, and butylated hydroxytoluene were obtained from Sigma (Roedermark, Germany). Internal standard reference [2H3]-3-O-Methyl quercetin was purchased from IsoReag (Shanghai, China).

The used instruments included hundred thousandth electronic analytical balance (Sartorius Practum, SQP, Sartorius scientific instruments (Beijing) Co., Ltd., Beijing, China), Milli-Q Plus machine (Millipore, Brussels, Belgium); vortex mixers (Vortex-Genie2, Scientific Industries, Bohemia, NY, USA); SpeedVac (Genevac miVac, Tegent Scientific Ltd., Oxford, UK).

#### 2.3.2. Metabolite Extraction

The extraction method was referenced in [23]. Briefly, 20 mg of sample was added with 0.8 mL of 90% methanol containing 0.5% acetic acid and 0.05% butylated hydroxytoluene, and incubated for 30 min at 1500 rpm and 4 °C; then, it was centrifuged for 10 min at 12,000 rpm and 4 °C. The supernatant was removed, placed into a clean 1.5 mL centrifuge tube, and dried using the SpeedVac; the dried extracts were redissolved in 0.5 mL 2% acetonitrile in water, 50 μL upper layer liquids mixed with 10 μL 0.5 μg/mL internal standard reference [2H3]-3-O-Methyl quercetin were obtained for LC-MS/MS analysis.

#### 2.3.3. LC-MS/MS Analysis

The LC-MS/MS analysis was carried out by Lipidall Technologies Company Limited, Changzhou, China. Multiple-reaction-monitoring (MRM) method was used for analyses of the metabolites. All instrumentation conditions and MRM parameters used for method validation were based on [23]. The column ACQUITY UPLC HSS T3 1.8 μm, 2.1 × 100 mm columns and a guard column (Waters, Dublin, Ireland) were equipped in the present research. High-performance liquid chromatography (Jasper, AB Sciex, Singapore) coupled to TripleQuad MS (4500 TripleQuad, AB Sciex, Singapore) was utilized to acquire metabolites data. The injector volume was 5 L. The flow rate was 0.40 mL/min. Mobile phase A was water containing 0.1% FA (*v/v*), and mobile phase B was ACN. The following linear gradient was used: 0–1.0 min with 2% B, 1.0–6.0 min with 2–42% B, 6.0–8.0 min with 42–65% B, 8.0–10.0 min with 65–76% B, 10.0–11.0 min with 76–100% B, 11.0–14.0 min with 100–100% B.

The temperatures of the column and auto-sampler were maintained at 40 °C and 4 °C, respectively. The injection volume was 5 μL per run, and the flow rate was 0.4 mL/min. The MS parameters for detection were: ESI (-) source voltage −4.5 kV, and +5.5 KV for ESI (+); vaporizer temperature, 500 °C; drying gas (N_2_) pressure, 50 psi; nebulizer gas (N_2_) pressure, 50 psi; curtain gas (N_2_) pressure, 35 psi.

### 2.4. Statistical Analysis

Data acquisition and processing were performed using MultiQuant 3.0.2 (AB Sciex). SIMCA (version 14.1, Umetrics, Umea, Sweden) and SPSS (version 26.0, SPSS Inc., Chicago, IL, USA) are used for multivariate statistical analysis (principal component analysis (PCA) and OPLS-DA). An analysis of variance (ANOVA) was used to assess the significance of differences in stable isotope ratios and polyphenols and alkaloids content of partridge tea from different geographical sources at a *p* = 0.05 confidence level. The data were pre-processed using unit variance scaling (UV) before modeling. The characteristics of polyphenols and alkaloids in partridge tea from different regions were analyzed by heat map analysis and hierarchical cluster analysis (HCA), in order to find the relatively homogeneous clusters in samples and explain the changes of metabolites in different groups of samples. For OPLS-DA models, R^2^ and Q^2^ are typically used to evaluate the quality and reliability of these models. Generally speaking, their values close to 1.0 indicate that the model has good adaptability and prediction ability [24].

The VIP score reflects the importance of the X variable relative to the Y variable in the model (the predictive part of the model) and can be used as a potential variable selection method to select the most differential geographically derived dependent metabolites. In general, the variable with VIP value greater than 1 is usually selected as an important index of the model [3,7,25,26]. So, according to VIP value in OPLS-DA analysis (VIP > 1), the meaningful compounds were screened out. For polyphenols and alkaloids, the random forest model was also established to study in depth the importance of the arrangement accuracy of these differential metabolic characteristics and further refine the marker compounds that could distinguish partridge tea from the four origins. In OPLS-DA analyses, SIMCA’s default analysis of variance based on 7-fold cross-validation prediction residuals were used to assess the statistical significance of the model. The random forest model is implemented by Python machine learning library Scikit-learn, and the parameters are set by default.

## 3. Results and Discussion

### 3.1. C/N/O/H Stable Isotope Analysis

**Figure 2:**
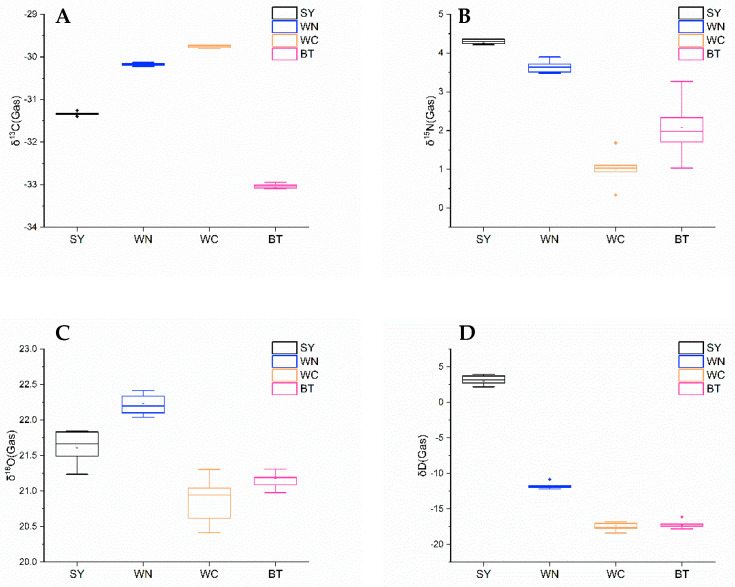
Box plots of δ^13^C (**A**), δ^15^N (**B**), δ^18^O (**C**), and δ D (**D**) of partridge tea from four geographical origins (SY, WN, WC, and BT). In the boxplot, outliers are indicated by asterisks. Outliers are data values that are remote from other data values.

The stable isotope ratios of C/N/O/H in SY, WN, WC, and BT regions were determined by EA-IRMS, and the results are summarized in Table 1. According to the ANOVA, it was judged whether there were significant differences (*p* < 0.05) in the C/N/O/H stable isotope ratios of partridge tea from four different geographical origins, which was indicated by the superscripts with different letters in Table 1. The distribution and variation of δ^13^C, δ^15^N, δ D, and δ^18^O of partridge tea from different geographical origins were also shown in the box plots (Figure 2).

The average δ^13^C values of all the samples were within the range of −29.75 to −33.04‰, with little fluctuation in each group of samples. The average δ^13^C value of the sample from WC was the highest (−29.75‰), followed by that of WN, SY and BT. According to the results of ANOVA based on δ^13^C values, there were significant differences (*p* < 0.05) among the four samples from different origins, indicating that δ^13^C values were effective indicators for distinguishing the samples of partridge tea from four different origins. The main factors affecting δ^13^C value were plant type and environmental factors (temperature, humidity, altitude, etc.), and the former had the most significant effect on δ^13^C value. Tea was a typical C3 plant (δ^13^C values from −25.0 to −35.0‰) [27], which was consistent with the δ^13^C values (−29.75 to −33.04‰) obtained in the present study.

The average δ^15^N value of all samples were within that range of 1.02–4.29‰, which was relatively consistent with the previously report δ^15^N range of Chinese green tea (1.8–4.6‰) [9]. δ^15^N values from BT samples showed the largest change, ranging from 1.03 to 3.27‰. δ^15^N values of SY samples were the highest, significantly higher than that of WC sample (the lowest). The δ^15^N values of SY and WN samples were close, and that of WC and BT samples were close. By ANOVA, there were significant differences among the four sample origins (*p* < 0.05), which confirmed the possibility of using δ^15^N value to test the geographical origin of partridge tea samples. This difference was greatly affected by the local planting pattern, fertilizer source, and application amount. In general, the range of δ^15^N value of synthetic or inorganic fertilizers was very narrow, close to 0‰, which was much lower than that of organic fertilizers [28].

In addition, the geographical and climatic conditions (such as precipitation, temperature, humidity, geographical location, and distance from the coast) are closely related to the changes in δ D and δ^18^O, and higher values of δ D and δ^18^O are reported in the rainfall near the coastal areas [29,30]. The average δ^18^O values of partridge tea from different origins in Hainan Province ranged from 20.88 to 22.22‰, the δ D value ranged from −17.54 to 3.16‰, and δ D values between the four origins fluctuated greatly. Compared with partridge tea from other regions, the samples from WN and SY showed the higher δ^18^O and δ D values, which might be due to the high annual precipitation, followed by BT and WC. There were significant differences (*p* < 0.05) in δ^18^O values of partridge tea from four origins. For δ D, the δ D values of SY is significantly higher than other regions, and the δ D values of WC and BT were close. There was no significant difference (*p* > 0.05) between the WC and BT regions, while there was significant difference (*p* < 0.05) between SY, WN and other two regions.

**Figure 3:**
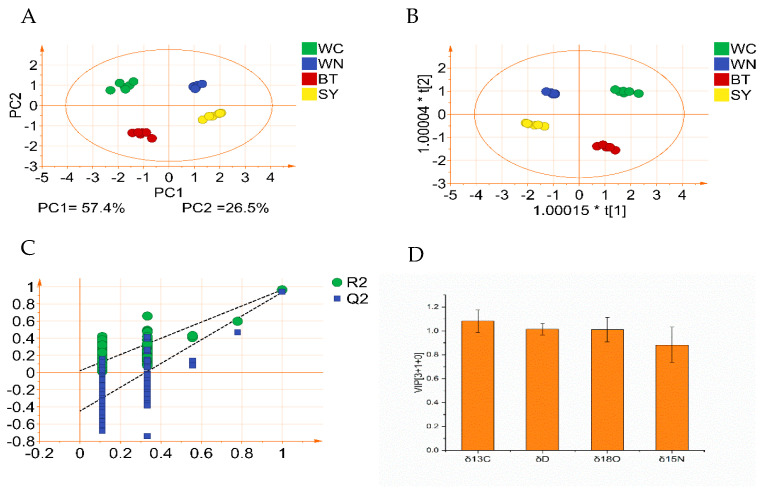
Principal component analysis (PCA) and orthogonal partial least squares discriminant analysis (OPLS-DA) results of stable isotope ratios of partridge tea from four origins (SY, WN, WC, and BT): (**A**) 2D scores plot of PCA; (**B**) 2D scores plot of OPLS-DA; (**C**) result map of 200 cross-validation of OPLS-DA model; (**D**) VIP map of contribution of each variable to sample classification.

According to the results of ANOVA, the C/N/O/H stable isotopes of partridge tea from different origins had significant differences. In order to preliminarily explore that data characteristics of C/N/O/H stable isotopes, an exploratory PCA was used [31]. Figure 3A shows the PCA 2D scores plot of the stable isotope ratios of partridge tea. The samples were clustered mainly based on their biological repetitions in the PCA scores plot, representing the partridge tea samples from SY, WN, WC, and BT, respectively. It could be seen that the samples were divided into four independent clusters. The first two principal components accounted for most of the variables (83.8%), and PC1 and PC2 accounted for 57.4% and 26.5%, respectively. The differentiation between the four samples was significant, indicating that partridge tea from four origins could be clustered based on the C/N/O/H stable isotope without considering the geographical origin.

In order to exclude intra-group differences, highlight inter-group differences, and identify the stable isotope differences of partridge tea caused by different origins, the OPLS-DA model based on the stable isotope ratio was further established. It could be clearly seen from the 2D scores plot of OPLS-DA (Figure 3B) that the partridge tea samples were divided into four clusters, with the high mutation explanation rate (R^2^X = 1, R^2^Y = 0.971) and high prediction ability (Q^2^ = 0.949), and the cross-validation classification accuracy rate was 100%. This indicated that the stable isotope ratio could be used as an effective index to distinguish partridge tea from different origins. In order to avoid over-fitting of the model, the OPLS-DA model was cross-validated with 200 permutation tests [32]. The results showed that the intercept of R^2^ and Q^2^ are −0.0215 and −0.449, respectively, which indicated that the model has good robustness (Figure 3C). The intercept of R^2^ and Q^2^ should be less than 0.30 and 0.05, respectively [33]. To assess the overall impact of the variables on the sample set differences, a projected VIP score plot for each variable was also generated. It can be seen from Figure 3D that the VIP value of δ^13^C, δ D, and δ^18^O is greater than 1, indicating that δ^13^C, δ D and δ^18^O have a significant contribution to the sample classification.

### 3.2. Targeted Metabolomics Fingerprinting Analysis

**Figure 4:**
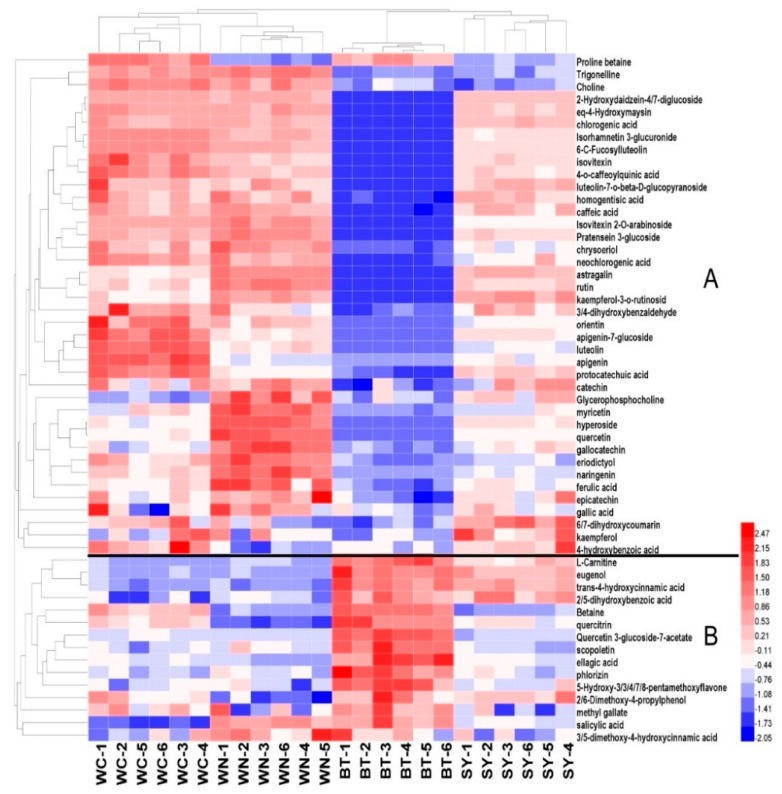
Heat map and hierarchical clustering analysis (HCA) of tea polyphenol and alkaloid contents of Hainan partridge tea from four origins (SY, WN, WC, and BT). According to the HCA diagram, the metabolites can be divided into two categories: (**A**,**B**).

A total of 55 metabolites (Including 49 polyphenols and six alkaloids) in partridge tea were detected by LC-MS/MS. The total ion chromatography (TIC) was shown in Figure S1. The data of 55 metabolites were standardized by SPSS using Z-score method, and the standardized data were plotted into heat map and HCA, in order to visualize the relationship between the metabolites of partridge tea from four regions (Figure 4). The heat map represented the rows of data for each metabolite in each column variable as a color block, with red boxes for higher levels and blue boxes for metabolites at lower levels. HCA is a clustering method that explores the grouping of samples through subsequent data aggregation or partitioning [34]. Catechins are the most important bioactive components in tea polyphenols; generally speaking, catechins are the main components of tea polyphenols [20]. However, in this study, gallic acid and ellagic acid in partridge tea polyphenols account for a large proportion, while catechins are relatively low; this is also consistent with previous reports that partridge tea is rich in ellagic acid and gallic acid [35]. The good aroma of partridge tea may be due to its high phenolic acid content, because phenolic acids are considered to be related to the aroma quality of tea [36,37].

Through heat map and HCA (Figure 4), 55 species metabolites were divided into two categories: Group A (40 species) and Group B (15 species). Group A represented the majority of polyphenols (36 species) and alkaloids (four species) in partridge tea, including all detected catechins, most phenolic acids, and most alkaloids. It could be seen from Figure 4 that the contents of WC, WN, and SY metabolites were all at high levels, which was significantly distinguished from BT. Among them, the contents of WN and WC were the highest, which might be the main reason for the good quality of WN and WC partridge tea, because higher polyphenol and alkaloid contents often represented higher tea quality. Among the metabolites belonging to Group B, the samples of BT were significantly higher than those of the other three regions. Group B included 15 metabolites, such as L-Carnitine, eugenol, trans-4-hydroxycinnamic acid, 2,5-dihydroxybenzoic acid, Betain, quercitrin, Quercetin 3-glucoside-7-acetate, scopoletin, ellagic acid, phlorizin, 5-Hydroxy-3,3,4,7,8-pentamethoxyflavone, 2,6-Dimethoxy-4-propylphenol, methyl gallate, salicylic acid, and 3,5-dimethoxy-4-hydroxycinnamic acid. These metabolites can be used as the main components to distinguish BT from the other three geographic origins of partridge tea. Ellagic acid was the highest content detected, and the ellagic acid content of BT sample was significantly higher than that in other regions. Ellagic acid has strong antioxidant and anticancer effects on human health [38].

In addition, as can be seen from the HCA figure at the top of Figure 4, partridge tea from different origins can be accurately divided into four categories by HCA, according to the metabolic maps of polyphenols and alkaloids. This indicated that the differences of polyphenols and alkaloids in partridge tea from different origins were significant. Further, the unsupervised PCA method was used to summarize the phenotypic differences of metabolites and the supervised OPLS-DA method was used to establish the prediction model.

**Figure 5:**
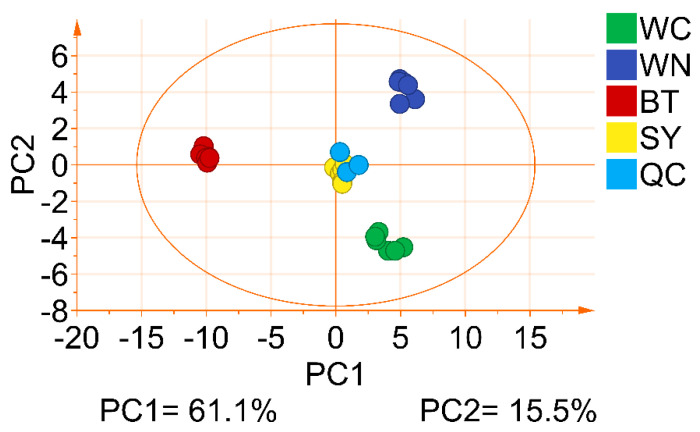
2D scores plot of the first two principal components based on PCA of polyphenols and alkaloids content, used for identifying partridge tea from four origins (SY, WN, WC and BT).

In order to evaluate the differences between polyphenols and alkaloids of partridge tea from different origins, the PCA based on polyphenols and alkaloids data was used. Figure 5 shows the 2D scores plot generated after PCA analysis, with the first two principal components accounting for most of the variables, and PC1 and PC2 accounting for 61.1% and 15.5%, respectively. The interpretation rate (R^2^X (cumulative) = 0.888) were satisfactory. It could be observed that the 2D scores plot of PCA divided the samples into four independent clusters, representing the partridge tea samples derived from SY, WN, WC, and BT, indicating that there were significant differences in the polyphenol and alkaloid metabolism spectra of partridge tea from different origins, and the samples could be naturally grouped according to their metabolite characteristics, which was consistent with the result obtained by HCA before. In addition, QC samples were used to verify the reliability of the data. The PCA scores plot showed tight clustering between the QC samples, with the QC samples positioned near the origin of the coordinates, which clearly demonstrated the stability and repeatability of the analytical method.

Although HCA and PCA could visually show how the samples were clustered, indicating a strong trend for identification of partridge tea from different origins by polyphenol and alkaloid contents, they could not provide information on clustering quality and clustering confidence [7]. Therefore, OPLS-DA analysis was further performed to help understand inter-class separation and identify potential categorical variables.

**Figure 6:**
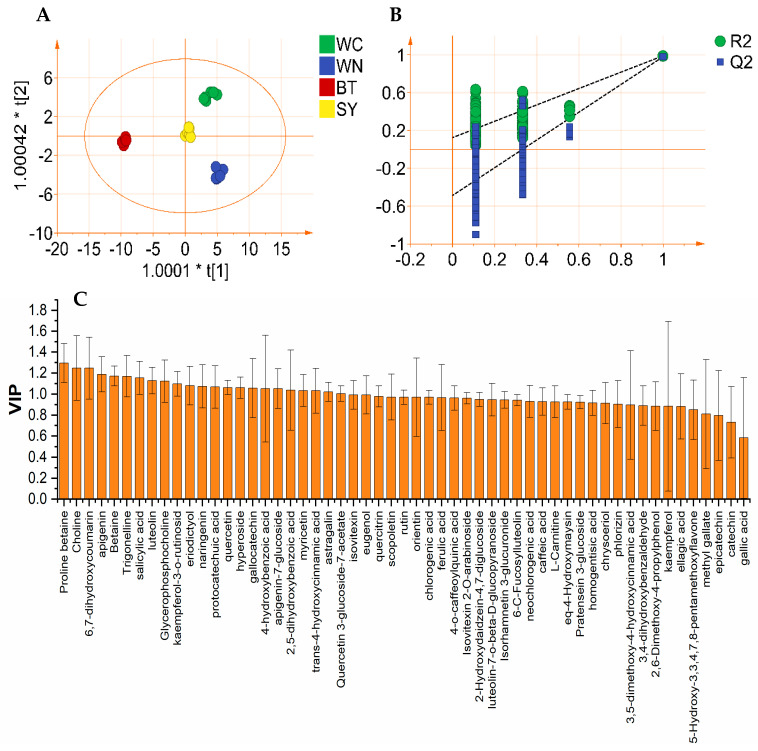
OPLS-DA results of polyphenol and alkaloid content of partridge tea from four origins (SY, WN, WC, and BT): (**A**) 2D scores plot of OPLS-DA; (**B**) result map of 200 cross-validation of OPLS-DA model; (**C**) VIP map of contribution of each variable to sample classification.

Figure 6 is the OPLS-DA analysis result based on polyphenols and alkaloids of partridge tea. It can be seen from the OPLS-DA 2D scores plot that the samples were divided into four categories, and the differences were significant (Figure 6A). The model can explain 86.3% of the variation (R^2^X = 0.863, R^2^Y = 0.985) and has high prediction ability (Q^2^ = 0.974). Cross-validation with 200 permutation tests showed that the OPLS-DA model has good robustness (intercept of R^2^ and Q^2^ are 0.123 and −0.488, respectively) (Figure 6B). Furthermore, in order to identify variables that contribute significantly to sample classification, a variable importance map in the predicted values was generated (Figure 6C) to evaluate the overall effect of the variables on the differences in sample set. The variables were screened according to VIP > 1 and *p* < 0.05, and 23 differential metabolites were finally screened out, as shown in Table 2. Five alkaloids were all included, indicating that the alkaloids of partridge tea might have significant potential for distinguishing partridge tea from different origins.

**Figure 7:**
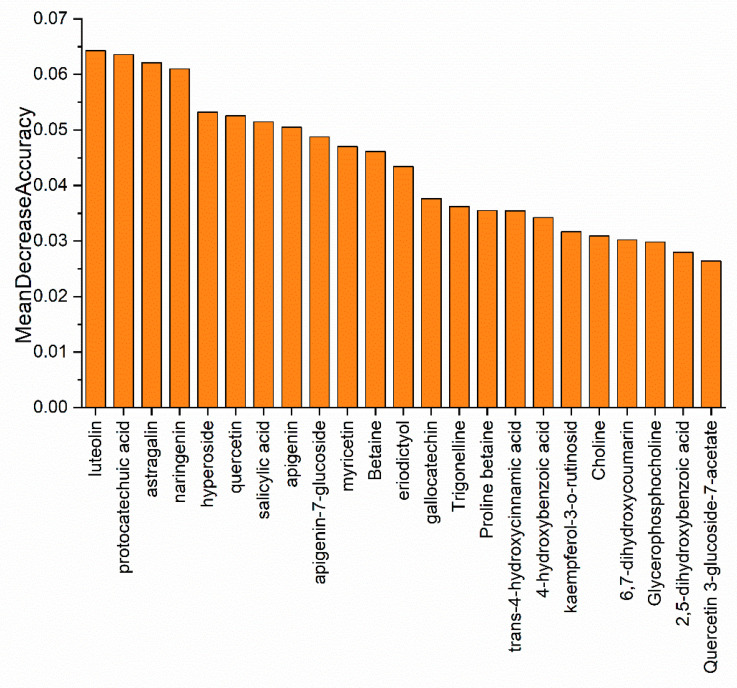
The relative importance of each variable in the random forest model and the importance of each variable is evaluated by the method of mean decrease accuracy.

OPLS-DA helped us to eliminate most of the variables that contributed less to the classification. The screened 23 differential metabolic characteristics could assist us in screening marker metabolites. However, due to the complexity of metabolite detection methods, we prefer to use as few variables as possible to achieve the purpose of identification. In addition, the data scaling method is an important influencing factor of OPLS-DA model, which may interfere with the selection of important variables in the model, and random forest is not sensitive to the selection of data scaling method, which is also a prominent advantage of random forest [39]. Based on the metabolic characteristics screened out by OPLS-DA, the random forest model was used for further screening, which could eliminate the interference to a certain extent. Therefore, the application of the decision combination of OPLS-DA and random forest in this study allowed us to observe the data from different angles, thereby accurately further refining the differential metabolic characteristics. Random forest is a combined classification model composed of multiple decision tree classification models. The optimal classification result is selected through the voting of multiple decision tree classification models. The importance of each variable is evaluated with the mean decreasing precision method. The parameters of the random forest model were set as the default settings. In order to improve the accuracy and stability of the feature selection, the modeling was repeated 100 times. The obtained variable importance results were ranked after the average value was taken, and the results were presented in the form of histogram (Figure 7). Then, the variables that showed greater feature importance in the modeling were selected to sequentially establish the random forest classification model, so as to find the minimum number of variables when the highest classification accuracy is reached. Finally, when the first four metabolite profiles (luteolin, protocatechuic acid, astragalin, and naringenin) were used to model the random forest, the out-of-bag (OOB) error of the model was 0.0, and the accuracy of cross-test prediction was 100%. This indicated that the four compounds represented sufficient traceability information and had practical application value for the identification of partridge tea origin from four geographical origins. The extracted ion chromatographs (XIC) of luteolin, protocatechuic acid, astragalin, and naringenin were shown in Figures S2–S5.

## 4. Conclusions

In this study, the C/N/O/H stable isotope ratios and polyphenol and alkaloid contents of partridge tea from SY, WN, WC, and BT regions in Hainan Province of China were determined, and these characteristics were used for the first time to identify the geographic origin of partridge tea. There were significant differences in the C/N/O/H stable isotope ratios and the contents of polyphenols and alkaloids among the partridge teas from the four regions, which indicated that the geographical origin had a greater impact on these indicators of partridge tea, and thus the geographical origin of partridge tea could be correctly identified. Through chemometric analysis, it was found that both the stable isotope ratio and polyphenol and alkaloid content can accurately classify the sample sources. Potential markers distinguishing the origin of partridge tea were identified, including three stable isotope ratios (δ^13^C, δ D, and δ^18^O) and four polyphenol characteristics (luteolin, protocatechuic acid, astragalin, and naringenin). Therefore, stable isotope ratio and polyphenol and alkaloid contents were effective indicators for identifying the origin of partridge tea, which would provide the basis for the origin tracing of partridge tea. The results of this study may also indicate that, in future studies, if a single detection method cannot distinguish samples from different origins, the combination of stable isotope and metabolomics methods may be a better method, because both of them have satisfactory results for origin identification.

## Figures and Tables

**Table 1:** The δ^13^C, δ^15^N, δ^18^O, and δ D values of Hainan partridge tea samples from four origins (SY, WN, WC, and BT). Superscripts on different letters indicate significant differences between variables.

Geographical Origin	δ¹⁵N (‰)		δ¹³C (‰)		δ¹⁸O (‰)		δ D (‰)	
	Range	Mean ± SD	Range	Mean ± SD	Range	Mean ± SD	Range	Mean ± SD
SY	4.21–4.36	4.29 ± 0.063 ^a^	−31.40–(−31.26)	−31.33 ± 0.044 ^c^	21.24–21.85	21.62 ± 0.24 ^b^	2.19–3.96	3.16 ± 0.66 ^a^
WN	3.48–3.90	3.65 ± 0.16 ^b^	−30.23–(−30.13)	−30.18 ± 0.033 ^b^	22.04–22.42	22.22 ± 0.14 ^a^	−12.18–(−10.83)	−11.73 ± 0.48 ^b^
WC	0.34–1.68	1.02 ± 0.43 ^d^	−29.80–(−29.72)	−29.75 ± 0.034 ^a^	20.41–21.30	20.88 ± 0.32 ^d^	−18.41–(−16.82)	−17.54 ± 0.56 ^c^
BT	1.03–3.27	2.05 ± 0.74 ^c^	−33.09–(−32.94)	−33.04 ± 0.056 ^d^	20.98–21.31	21.16 ± 0.11 ^c^	−17.81–(−16.12)	−17.11 ± 0.56 ^c^

**Table 2:** Differential metabolites of partridge tea from four origins (SY, WN, WC, and BT) (based on VIP > 1 and *p* < 0.05).

NO.	Compound	Classification	VIP	*p*
1	Proline betaine	alkaloid	1.29654	<0.01
2	Choline	alkaloid	1.24792	<0.01
3	6,7-dihydroxycoumarin	Polyphenol	1.24765	<0.01
4	apigenin	Polyphenol	1.18786	<0.01
5	Betaine	alkaloid	1.17243	<0.01
6	Trigonelline	alkaloid	1.16975	<0.01
7	salicylic acid	Polyphenol	1.15381	<0.01
8	luteolin	Polyphenol	1.12937	<0.01
9	Glycerophosphocholine	alkaloid	1.12302	<0.01
10	kaempferol-3-o-rutinosid	Polyphenol	1.09786	<0.01
11	eriodictyol	Polyphenol	1.08076	<0.01
12	naringenin	Polyphenol	1.07391	<0.01
13	protocatechuic acid	Polyphenol	1.06823	<0.01
14	quercetin	Polyphenol	1.06197	<0.01
15	hyperoside	Polyphenol	1.06062	<0.01
16	gallocatechin	Polyphenol	1.05693	<0.01
17	4-hydroxybenzoic acid	Polyphenol	1.05198	<0.01
18	apigenin-7-glucoside	Polyphenol	1.05169	<0.01
19	2,5-dihydroxybenzoic acid	Polyphenol	1.03708	<0.01
20	myricetin	Polyphenol	1.03414	<0.01
21	trans-4-hydroxycinnamic acid	Polyphenol	1.0322	<0.01
22	astragalin	Polyphenol	1.02076	<0.01
23	Quercetin 3-glucoside-7-acetate	Polyphenol	1.00454	<0.01

## Data Availability

No new data were created or analyzed in this study. Data sharing is not applicable to this article.

## Supplementary Materials

The following are available online at https://www.mdpi.com/article/10.3390/foods10092130/s1. Figure S1. Total ion chromatography (TIC) of Hainan partridge tea by LC-MS/MS. Figures S2–S5. Extracted ion chromatographs (XIC) of luteolin, protocatechuic acid, astragalin and naringenin.
