# Supplementary material for: Authentication of Geographical Origin in Hainan Partridge Tea (Mallotus obongifolius) by Stable Isotope and Targeted Metabolomics Combined with Chemometrics
Source: Foods. 2021 Sep 9;10(9):2130. doi: 10.3390/foods10092130 (PMC8464849; doi:10.3390/foods10092130)
Supplement: Supplementary file 1 [file foods-10-02130-s001.zip › foods-1341576-supplementary.pdf]

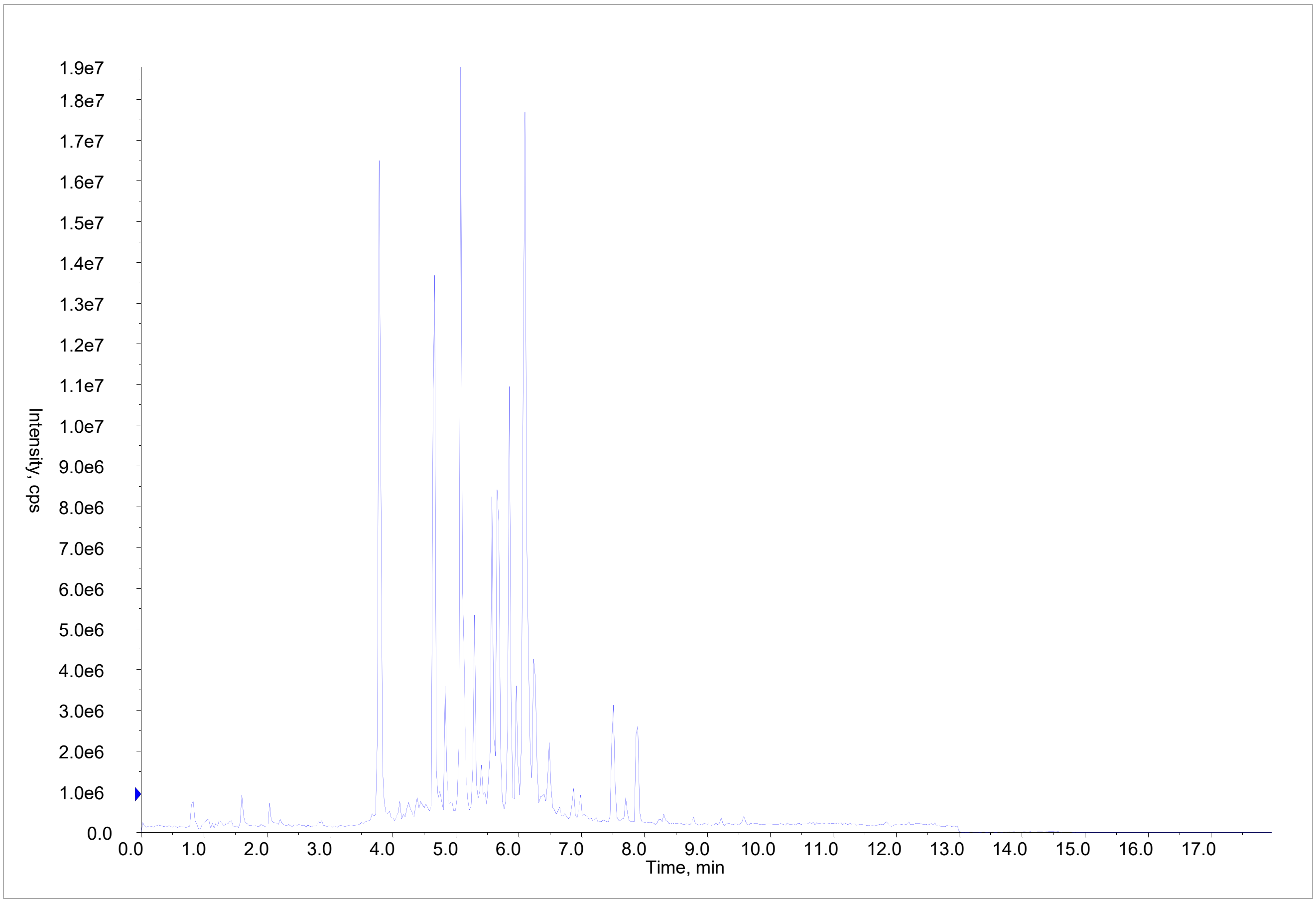

Figure S1. Total ion chromatography (TIC) of Hainan partridge tea by LC-MS/MS.

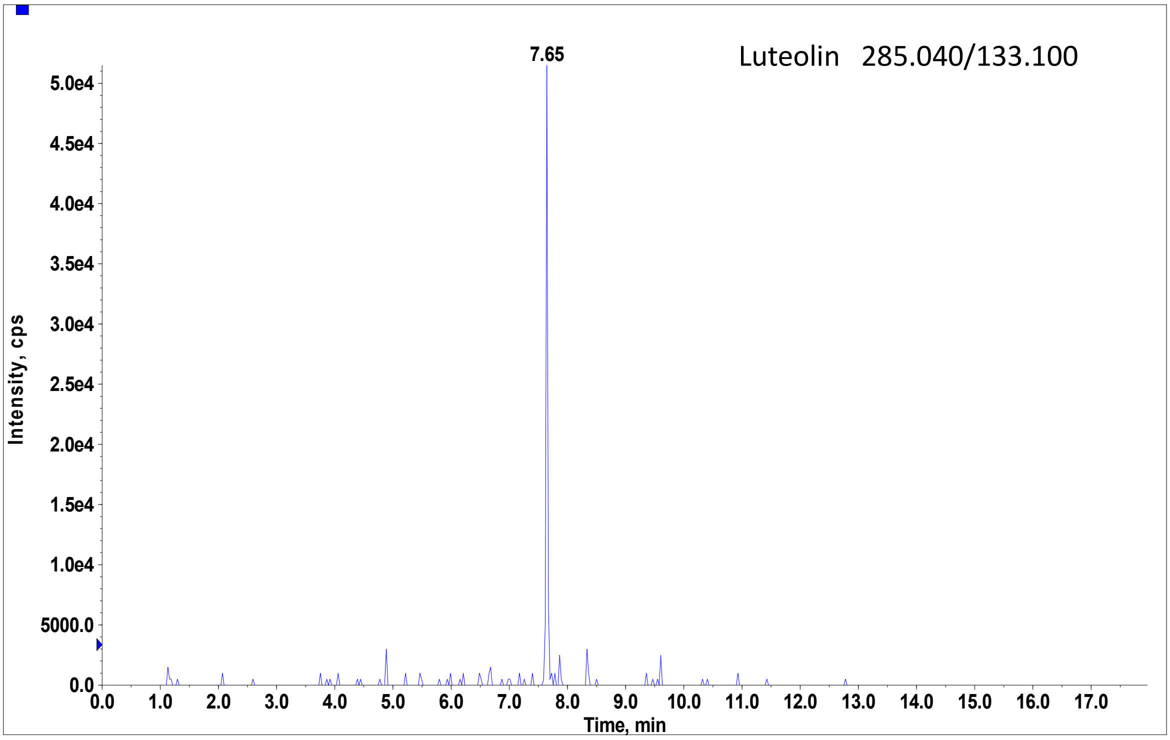

Figure S2. Extracted ion chromatography (XIC) of luteolin.

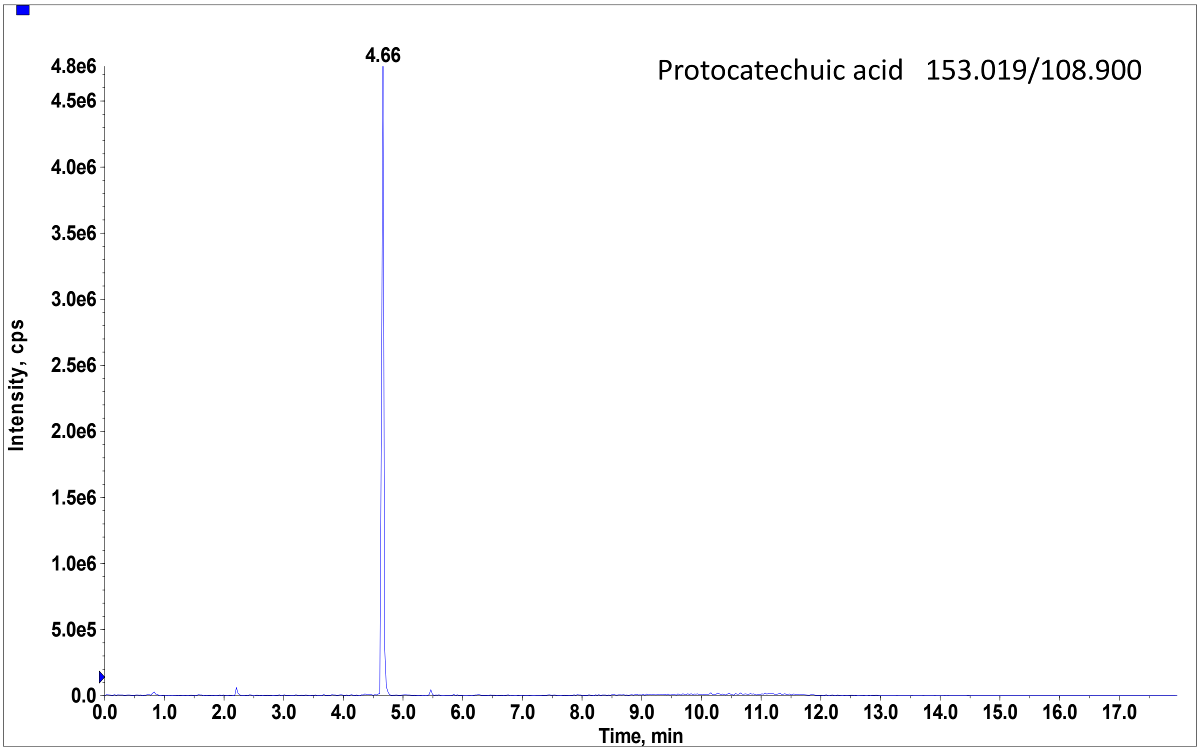

Figure S3. Extracted ion chromatography (XIC) of protocatechuic acid.

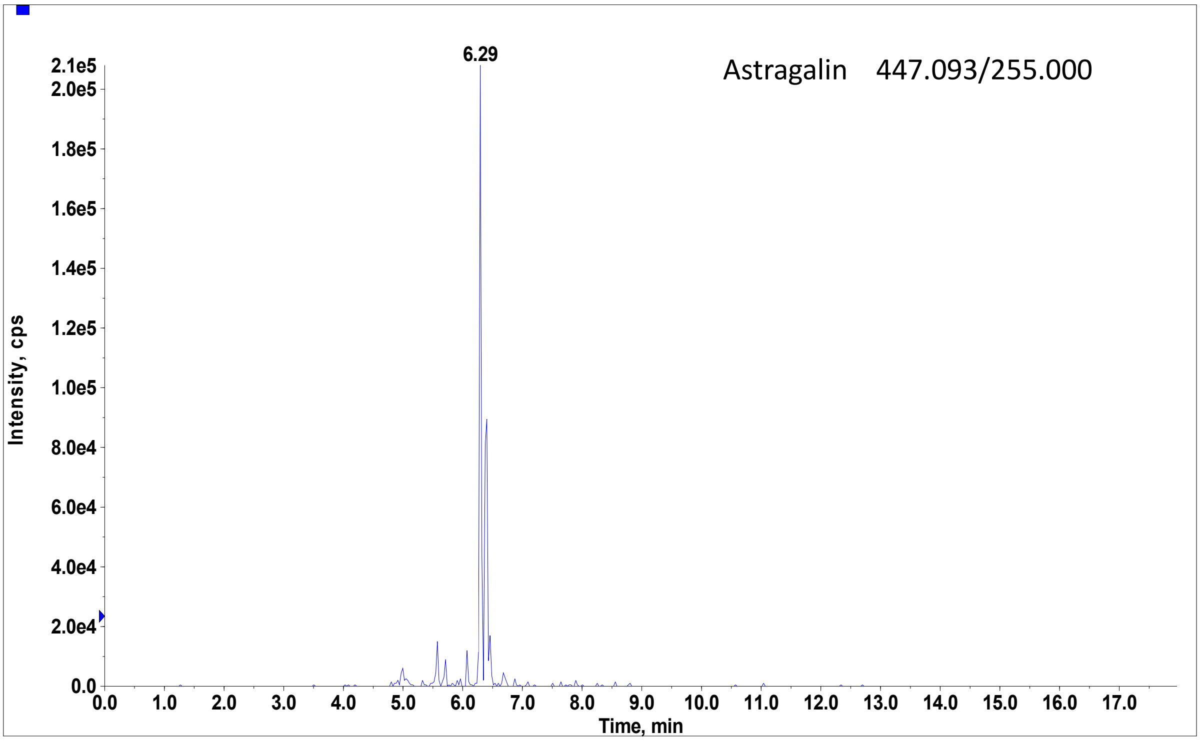

Figure S4. Extracted ion chromatography (XIC) of astragalin.

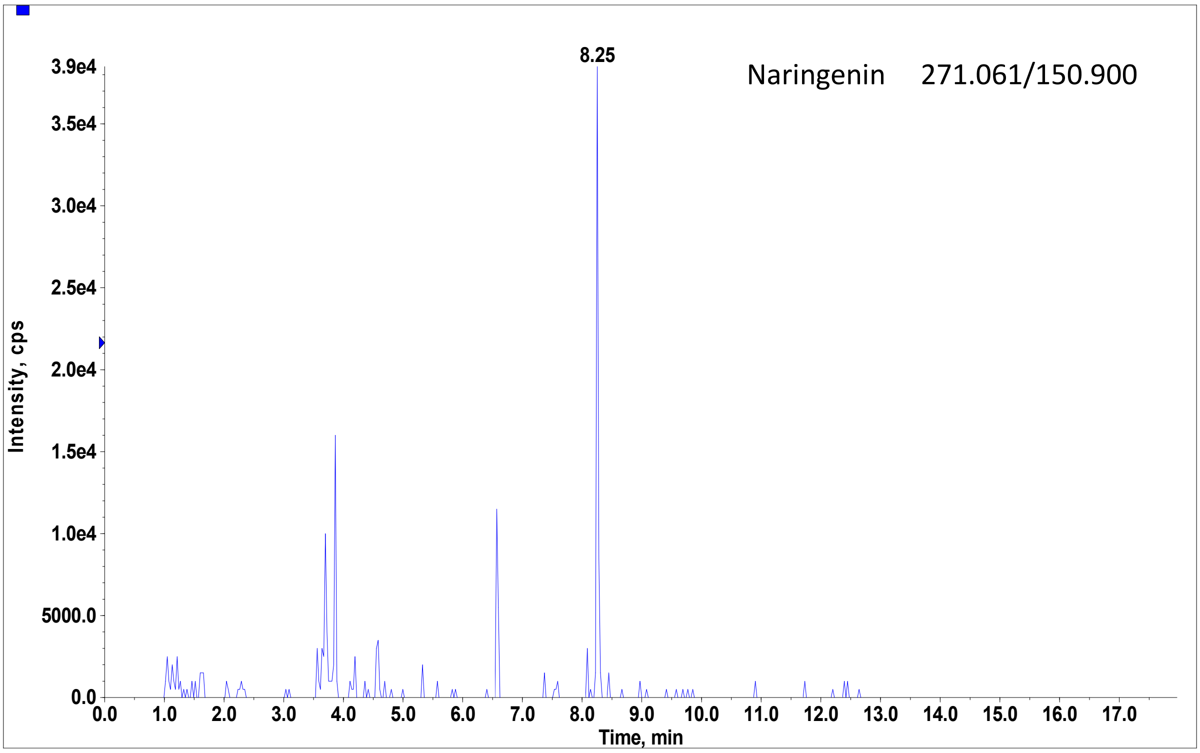

Figure S5. Extracted ion chromatography (XIC) of Naringenin.
